# Network Collapse and Cognitive Impairment in Multiple Sclerosis

**DOI:** 10.3389/fneur.2015.00082

**Published:** 2015-04-14

**Authors:** Menno M. Schoonheim, Kim A. Meijer, Jeroen J. G. Geurts

**Affiliations:** ^1^Department of Anatomy and Neurosciences, Neuroscience Campus Amsterdam, VU University Medical Center, Amsterdam, Netherlands

**Keywords:** multiple sclerosis, cognition, connectivity, activation, networks, functional reorganization, functional MRI

## Functional Reorganization in MS: An Outdated Concept?

The current field of multiple sclerosis (MS) research is an active and highly interesting one: structural abnormalities such as inflammatory lesions and brain atrophy are studied with a wide array of advanced neuroimaging techniques (1). These techniques are subsequently used to try to explain the large clinical heterogeneity in patients. Clinically important in MS is cognitive dysfunction, which is present in 40–70% of all patients (2, 3). Cognitive impairment in MS receives much attention, as there is currently no proven effective treatment, but symptoms may nevertheless start in early stages of disease already (4). Cognitive decline is known to exert deleterious effects on psychosocial functioning (2, 5, 6). Traditional structural imaging measures like lesion volumes are notoriously poorly related with cognitive function (7), so a move toward more sensitive, comprehensive measures is required, such as those that measure brain function in addition to brain structure.

Historically, most early imaging studies have used the paced auditory serial addition test (PASAT) to study cognition in MS, a task that measures information processing speed (8–10). These observed a combination of hyperactivation of frontal regions in response to the task and a recruitment of additional areas, not normally attributed to the task in controls. The functional changes were mostly positively related to the amount of structural damage in the brain, and were stronger in patients who scored normally on the PASAT, indicating that it might be a beneficial process. Later studies investigated other cognitive domains and also showed such an apparently beneficial increased local activation, for example, during a memory task in the hippocampus (11) and during the N-back working memory task in the dorsolateral prefrontal cortex (DLPFC) (12). Importantly, these studies also showed decreased activation in cognitively impaired patients.

The body of literature of that point in time led to our previous hypothesis of functional reorganization in MS (13). This hypothesis asserted that a “compensatory” change is seen in the brains of MS patients in the form of an increase in brain function, i.e., both increased activation and increased connectivity. Functional connectivity is conceptually quite different from task-based activation and reflects the amount of communication between brain regions, i.e., coherent patterns of firing typically measured with correlation measures. Early connectivity studies investigated the so-called “default mode network” (DMN), which is only coherently active during a resting state. Two such studies found DMN changes that were interpreted in the same way as the task-based activation studies: increased DMN connectivity in clinically isolated syndrome (CIS) patients (14) and decreased DMN connectivity in progressive MS, which was related to cognitive impairment (15). We proposed that increasing structural damage, in combination with an optimum curve of “functional reorganization,” results in a delayed, non-linear, development of cognitive dysfunction.

However, the previous model was mostly based on task-based activation studies, while the connectivity field was still in its infancy. As the concept of functional reorganization was gaining support, the field was primed for finding cognitively relevant connectivity changes. Interestingly, recent studies have mostly related increased functional connectivity to cognitive dysfunction, raising doubts on the previous concept of functional reorganization in MS. In this paper, we will review this recent functional connectivity literature and reiterate the case around functional connectivity changes in MS and their potential effects on cognition. Which reported connectivity changes can be justifiably said to be “compensatory” or “beneficial”? Which are likely “maladaptive”? Can any such predicate be arrived at all, based on the neuroscientific studies available? Is it perhaps time to revise our previous model of functional reorganization?

## Functional Connectivity in MS: A Field of Contradictions

Resting state network changes have been observed in relapsing remitting MS (RRMS) patients, both within and between almost all resting state sub-networks (16). The DMN de-activates when performing a task, and appears to be strongly related to cognition. DMN changes have been difficult to place within our previous hypothesis, as cognitive dysfunction was related to both decreased (17–21) and increased DMN connectivity (22–24). In pediatric MS, increased DMN connectivity was seen in cognitively preserved patients in the anterior cingulate gyrus, while decreased connectivity of the posterior cingulate was seen in cognitively impaired patients (25). Increased connectivity of the anterior cingulate cortex was also found in adult MS patients, although these connectivity changes showed both positive and negative correlations with cognitive dysfunction (26). Another recent paper in adult-onset MS suggests that the severity of cognitive impairment is directly related to the level of increased functional connectivity of the DMN (27). As the DMN de-activates during tasks, task-based studies have also looked at this network. During performance of the N-back working memory task, researchers noted less de-activation of the DMN (12) in cognitively impaired patients. Another recent study, however, seems to contradict this finding, as an increased DMN activation during a similar task was related to both higher intellectual enrichment and information processing speed performance (28). In short, the DMN results have been difficult to interpret.

Unfortunately, results from seed-based analyses investigating other structures like the DLPFC have not been very consistent either. One such study (29) found a reduced connectivity between the DLPFC and the superior medial frontal gyrus in patients who scored normally on the N-back, in relation to increased difficulty of the task, and also found increased connectivity between the left and right prefrontal cortices. This connectivity between the DLPFC and medial frontal regions was increased in MS patients in another study, during the Go/No Go task, at which they were impaired (30). The DLPFC was also studied during performance of the PASAT in patients with CIS who were impaired on this test (31, 32), showing decreased connectivity with several areas, including the anterior cingulate and thalamus. Contrarily, another study only showed increased connectivity during the PASAT in CIS patients, who were also impaired on this test (33).

Studies looking at several other cognitively relevant structures such as the thalamus, hippocampus, and cerebellum have shown varying patterns of connectivity in MS as well. Thalamic atrophy has well-known and strong effects on cognition in MS (34), which appears related to global cortical network changes (24, 35). An aforementioned task-based CIS study showed decreased connectivity between the thalamus and DLPFC during the PASAT (31), at which patients were impaired. Strikingly, during a resting state, the thalamus has also been shown to have increased connectivity with frontal areas in clinically definite MS patients with cognitive impairment (36, 37). Similarly, at rest, the hippocampus showed decreased connectivity related to hippocampal atrophy in patients with still intact memory performance (38), but increased connectivity in patients with memory impairment (39). The cerebellum, however, showed decreased connectivity in patients with cognitive dysfunction, both during the PASAT (40) and Stroop tasks (41).

## What Does it all Mean?

As described above, the body of literature on cognitively relevant connectivity changes in MS is currently difficult to interpret. As it seems, our previous model for functional reorganization is incomplete and the term is currently used in a number of ways and lacks a clear definition. Additionally, these findings were studied across the spectrum of clinical and cognitive phenotypes in MS, with very different methodological and statistical approaches, leaving the data ambiguous in places. Some studies now refer to any connectivity change as functional reorganization, leaving it to the reader to disentangle “beneficial” or “maladaptive” functional reorganization *post hoc*. This process actually seems quite complicated, however, as cross-sectional studies have related both connectivity increases and decreases to cognitive dysfunction in MS. Therefore, the studies that do claim that changes might be beneficial for cognitive performance in MS might not have enough evidence to do so. In truth, we are currently unable to disentangle “good” from “bad” and are strongly limited by the cross-sectional nature of almost all of these studies.

For example, suppose that a functional connectivity increase is observed in cognitively preserved patients, and a decrease in a cognitively impaired patient group. Although many studies interpret such a finding as cognitively relevant, as described previously, such data could, in fact, be interpreted in several ways. First, the functional connectivity increase in cognitively preserved patients might reflect “beneficial” functional reorganization, delaying cognitive impairment. In impaired patients, this effect of functional reorganization is then lost. Second, the functional connectivity increase in cognitively preserved patients might be a “maladaptive” response, following, e.g., disinhibition, heralding an imminent network collapse, and further deterioration into cognitive impairment. Third, the functional connectivity increase in cognitively preserved patients could be an unrelated epiphenomenon. Or, that the connectivity increase is related to structural damage, but that it has no direct impact on cognition at all. And finally, given the fact that most studies are cross-sectional, it cannot be excluded that the frequently observed functional connectivity increases in patients with cognitive impairment are, in fact, “beneficial.” It is possible that such increases are, e.g., a bleed through of beneficial functional reorganization from the cognitively preserved stage. This could be due to a poor definition of cognitive impairment and/or plastic changes that persist throughout this stage of the disease. The only way we are going to understand the cognitive role of functional connectivity changes in MS will be to study them over time.

Preliminary longitudinal studies linking connectivity changes to cognitive rehabilitation (42, 43), as well as pharmacological intervention (44), show some promise. Unfortunately, determining sufficient sample sizes and time frames remains difficult given the current lack of data, leaving these small studies difficult to interpret. Such intervention studies aiming to increase neurotransmitter levels in MS appear logical, as there is an apparent cholinergic (45) as well as glutamate (46) imbalance in MS, which might leave the network unstable. Therefore, pharmacological therapies targeting such neurotransmitters might prove valuable (47). It must be stressed, however, that there may also be downsides to such an approach, as specific glutamate receptor subtypes have been linked to brain atrophy (48) and excitotoxic effects due to the treatment and the functional reorganization process might actually increase tissue damage and network stress.

## The Future: Measuring Network Collapse in MS

**Figure 1:**
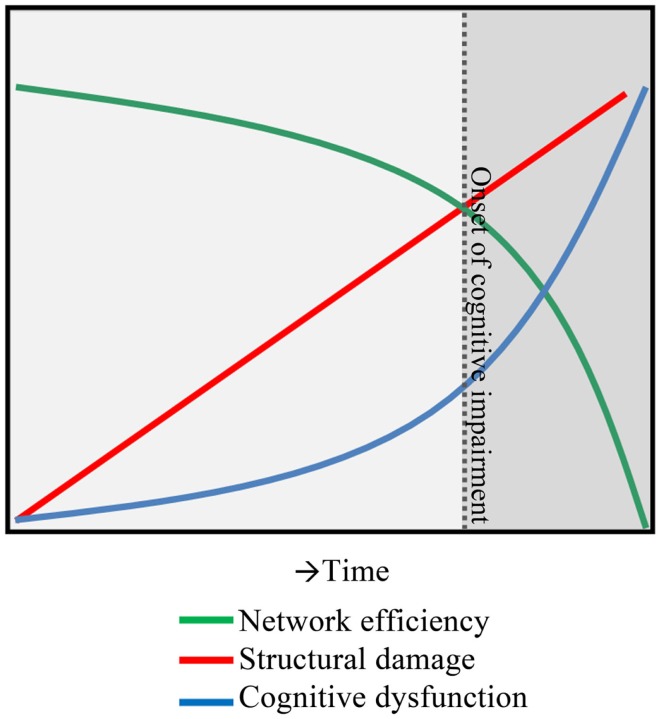
**A hypothesis of network collapse as a cause for developing cognitive impairment in MS**. In early stages of MS, structural damage is low, leaving network efficiency relatively high. As the structural damage accumulates over time, network efficiency levels drop, inducing a network collapse after a critical threshold (indicated by the dotted line) is exceeded. After this, the network is unable to function normally and cognitive impairment develops.

As the field of functional imaging in MS matured, the clinical interpretation of the *combined set* of functional changes in MS has become much more complex, leaving our previous model of functional reorganization in MS incomplete and too simplistic. After exploring abovementioned individual structures and sub-networks in MS has not made matters much clearer, it is now opportune to look at connectivity in another way. One option is to take functional connectivity values and convert them into a more holistic network model of the entire brain. This so-called graph analysis approach (49) uses different parameters such as the clustering coefficient and path length (50) to describe network information flow. Applications of these techniques in MS have been very limited (49), but have highlighted the power of graph analysis in discriminating patients from controls (51). Graph analytical studies in MS have shown that cognitive dysfunction is related to an inefficient network, as seen by the change in clustering coefficient and path length (52–54), impaired network integration of information (55) and clustering (56), decreases in network centrality (57, 58), increases in modularity (59), and changes in minimum spanning tree parameters (35, 60). These graph measures provide us many new ways to conceptualize and understand what actually happens to the global status of the entire brain network in patients with cognitive impairment in MS, beyond the poorly understood local increases or decreases in connectivity. Future longitudinal studies are now required to assess the predictive power of these measures. Together, it appears that the brain network of patients with cognitive impairment in MS features a strong decrease in whole-network *efficiency*, i.e., a network “collapse” (see Figure 1).

In summary, thinking about functional reorganization processes and labeling them as either “beneficial” or “maladaptive” has proven to be overly simplistic. A more holistic approach is required, encompassing both activation and connectivity data into a frame of network dynamics in a longitudinal fashion. Following this, first steps toward using more sophisticated (functional) imaging tools to monitor cognitive deficits can hopefully be taken.

## Author Contributions

All authors contributed to the conception, drafting, revising, and finalizing of the manuscript and agree to be accountable for all aspects of the work.

## Conflict of Interest Statement

The authors declare that the research was conducted in the absence of any commercial or financial relationships that could be construed as a potential conflict of interest.
